# Saikosaponin A and Its Epimers Alleviate LPS-Induced Acute Lung Injury in Mice

**DOI:** 10.3390/molecules28030967

**Published:** 2023-01-18

**Authors:** Donghui Peng, Yuchan Chen, Yanping Sun, Zhihong Zhang, Na Cui, Wensen Zhang, Ying Qi, Yuanning Zeng, Bin Hu, Bingyou Yang, Qiuhong Wang, Haixue Kuang

**Affiliations:** 1 Key Laboratory of Basic and Application Research of Beiyao, Ministry of Education, Heilongjiang University of Chinese Medicine, No. 24, Heping Road, Harbin 150040, China; 2 Guangdong Engineering Technology Research Center for Standardized Processing of Chinese Materia Medica, Science and Technology Department of Guangdong Province, Guangdong Pharmaceutical University, No. 280, Waihuan East Road, Guangzhou 510006, China; 3 National Engineering Research Center for Modernization of Traditional Chinese Medicine-Hakka Medical Resources Branch, School of Pharmacy, Gannan Medical University, No. 1, Medical College Road, Ganzhou 341004, China

**Keywords:** *Bupleurum chinense* DC., vinegar processing, saikosaponin A, saikosaponin b1, saikosaponin b2, saikosaponin D, acute lung injury

## Abstract

The purpose of this work was to illustrate the effect of processing with vinegar on saikosaponins of *Bupleurum chinense* DC. (BC) and the protective effects of saikosaponin A (SSA), saikosaponin b1 (SSb1), saikosaponin b2 (SSb2), and saikosaponin D (SSD) in lipopolysaccharide (LPS)-induced acute lung injury (ALI) mice. We comprehensively evaluated the anti-inflammatory effects and potential mechanisms of SSA, SSb1, SSb2, and SSD through an LPS-induced ALI model using intratracheal injection. The results showed that SSA, SSb1, SSb2, and SSD significantly decreased pulmonary edema; reduced the levels of IL-6, TNF-α, and IL-1β in serum and lung tissues; alleviated pulmonary pathological damage; and decreased the levels of the IL-6, TNF-α, and IL-1β genes and the expression of NF-κB/TLR4-related proteins. Interestingly, they were similar in structure, but SSb2 had a better anti-inflammatory effect at the same dose, according to a principal component analysis. These findings indicated that it may not have been comprehensive to only use SSA and SSD as indicators to evaluate the quality of BC, especially as the contents of SSb1 and SSb2 in vinegar-processed BC were significantly increased.

## 1. Introduction

Acute lung injury (ALI) is a disease with high mortality and morbidity that is characterized by inflammatory factor storms, neutrophil infiltration, lung edema, and diffuse alveolar damage [1]. ALI can be induced by many stimuli, including viral infections, drugs, drowning, trauma, ischemia reperfusion, and shock, the pathophysiological features of which include lung epithelial cell and endothelial cell injury, oxygenation disorder, and respiratory distress, and further aggravation may lead to acute respiratory distress syndrome (ARDS) [2,3,4]. Multiple studies have found that alveolar macrophages play an important role in the development of pulmonary inflammation and are the main sources of many inflammatory cytokines, such as tumor necrosis factor α (TNF-α), interleukin 1β (IL-1β), and interleukin 6 (IL-6). These inflammatory cytokines evoke further severe lung injuries [5,6,7]. Nuclear transcription factor kappa-B (NF-κB) has been confirmed to play an important role in lung injury, which regulates the production of inflammatory cytokines [8]. Studies have found that lung tissue injury, inflammatory scores, and antioxidant indices were relieved in LPS-induced ALI mice by inhibiting the NF-κB signaling pathway [9,10]. Therefore, an effective anti-inflammatory medicine for the treatment of ALI by inhibiting NF-κB protein is urgently required.

*Bupleurum chinense* DC. (BC) has been used in China for more than 2000 years as a common herb for relieving exterior syndrome, which was investigated to enter the liver, gallbladder, and lung meridians, and it has the functions of evacuation, reducing fever, soothing the liver, and relieving depression. BC mainly contains saikosaponins (SSs), volatile oil, polysaccharides, and flavonoid active components, and modern pharmacological research has found that BC has antipyretic, hepatoprotective, anti-inflammatory, antitumor, immunoregulation, and antidepressant effects [11,12]. Vinegar processing is the most common method of processing BC, which can strengthen the liver soothing and depression relieving effects. This is probably related to the changes in the SS content after processing with vinegar [13].

Because metabonomics has the characteristics of integrity, comprehensiveness, and dynamics, which coincide with the holistic view of traditional Chinese medicine, it has been widely used in the field of Chinese medicine processing in recent years. Raw and processed Astragali Radix could be obviously separated by LC-MS combined with multivariate statistical analysis, and 15 notable markers could be used to determine different processed drugs [14]. Consequently, in this study, ultra high performance liquid chromatography with orbital trap high-resolution mass spectrometry (UHPLC-Oribtrap/HRMS) combined with a multivariate statistical analysis was used to compare the different chemical constituents in BC and vinegar-processed BC (VBC) to determine the characteristic chemical markers of BC and vinegar processing products.

SSs are the main active components of BC, which has been confirmed to have antiviral, anti-inflammatory, antioxidant, antitumor, antifibrosis, antidepressant, and antiepileptic effects [15,16,17,18,19,20,21]. It has been proven that saikosaponin A (SSA) and saikosaponin D (SSD) have good anti-inflammatory effects, and they are also used as indicator components for BC quality evaluation in the Chinese Pharmacopoeia. SSA reduced lung pathological injury, the lung wet-to-dry (W/D) ratio, MPO activity, and inflammatory cytokines TNF-α and IL-1β in BALF in LPS-induced ALI mice. These results were closely related to the inhibition of the NF-κB and Nod-like receptor family protein 3 (NLRP3) signaling pathways [22]. SSD is a stereoisomer of SSA, which possesses anti-inflammatory and immune-modulatory properties. A study demonstrated that SSD may attenuate ventilator-induced lung injury through the inhibition of inflammatory responses, oxidative stress, and apoptosis [23]. Several studies showed that saikosaponin b2 (SSb2) attenuated kidney fibrosis, restrained breast cancer cell proliferation, enhanced the liver-targeting effect of anticancer drugs, and suppressed inflammatory responses in LPS-induced RAW 264.7 macrophages, but the protective effects of saikosaponin b1 (SSb1) and SSb2 in LPS-induced ALI mice have rarely been reported [24,25,26,27].

In this study, we explored the changes in the SS components of BC before and after being processed with vinegar based on UHPLC-Orbitrap/HRMS. Then, the anti-inflammatory effects and potential mechanisms of SSA, SSD, SSb1, and SSb2 in LPS-induced ALI mice were researched, and a principal component analysis was used to comprehensively evaluate their anti-inflammatory actions, which were intended to provide a scientific basis for fully clarifying the principles of BC processing as well as candidate drugs for improved treatment of ALI.

## 2. Results

### 2.1. Analysis of Bupleurum chinense DC. Methanol Extracts

**Figure 1:**
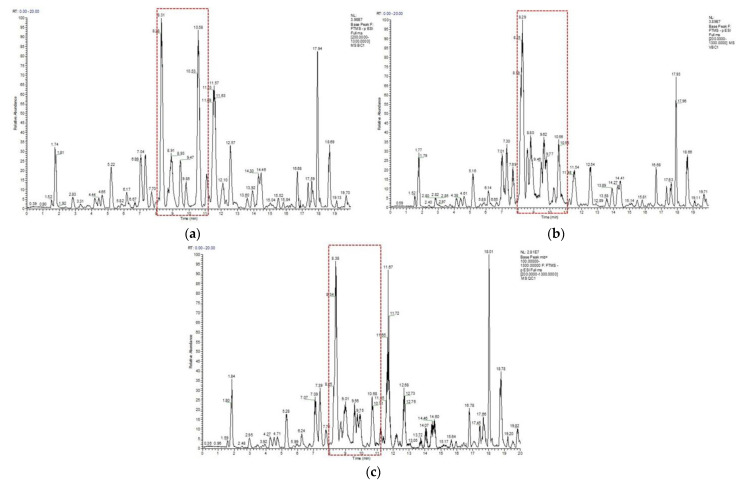
The representative total ion flow chromatograms of BC (**a**), VBC (**b**), and QC (**c**).

After optimizing the mass spectrometer parameters, the total ion flow chromatograms of BC and VBC extracts under the negative ion scanning mode were collected, as shown in Figure 1a–c. A total of fifty–two compounds were identified using a literature investigation, database search, fragmentation mode analysis, and standard comparison, including forty–five SSs, five flavonoids, one phenylpropanoid, and one lignan, as shown in Table 1 [28,29,30,31].

**Figure 2:**
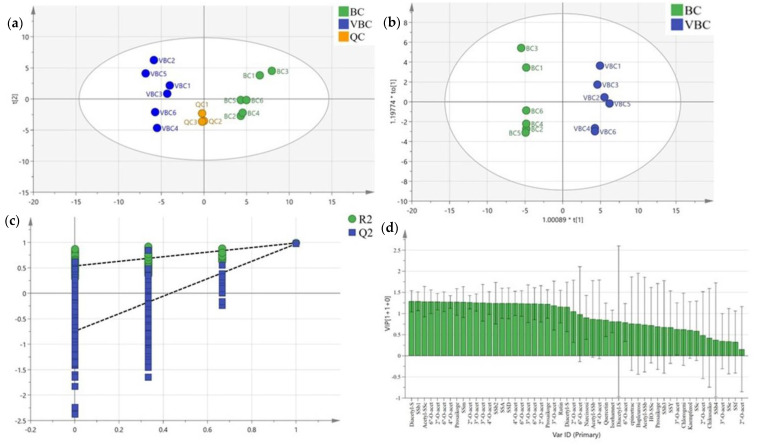
The results of a multivariate statistical analysis of BC and VBC methanol extracts: (**a**) PCA score plots; (**b**) OPLS−DA score plots; (**c**) OPLS−DA model validation plots; (**d**) VIP score plots.

The peak areas of the compounds identified in negative ion mode were imported into SIMCA 14.0 for the multivariate statistical analysis. The unsupervised principal components analysis (PCA) score scatter plot found a high aggregation of QC samples, which indicated that the instrument was stable and the data were reliable during the experiment. The distribution sites of the BC and VBC groups were obviously separated, which showed that the chemical composition of BC was obviously different from that of VBC. In particular, the metabolic profiles in the red boxes were significantly different between the two groups (Figure 1 and Figure 2a). 

**Figure 3:**
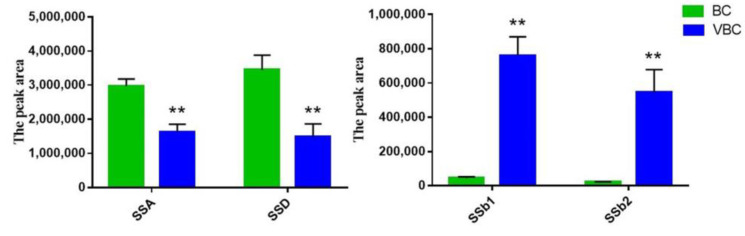
The peak area statistics of SSA, SSb1, SSb2, and SSD from the BC and VBC groups (*n* = 6, means ± SDs). Analyzed using a one-way ANOVA. ** *p* < 0.01: BC group vs. VBC group.

To better verify the difference in chemical composition between BC and VBC, an orthogonal partial least squares discriminant analysis (OPLS−DA) was performed, and the score plot model is exhibited in Figure 2b. The statistical parameters of the supervised OPLS−DA were R^2^X = 0.688, R^2^Y = 0.989, and Q^2^ = 0.971, which indicated that the model fitting results were acceptable. After carrying out permutation tests (permutation number: 200), the results showed that the crossover points between the Q^2^ regression line and the vertical axis were less than zero, which indicated that the models were not overfitted and the model verification was effective (Figure 2c). The variable importance on projection (VIP) represented the difference contributed to distinguishing two groups in the OPLS−DA model. Compounds with VIP scores greater than one were considered to be important variables to distinguish BC and VBC extracts. In this study, twenty−six compounds were optioned with VIP values greater than 1.0. Specific compound information is listed in Table 2 and Figure 2d. In addition, the peak area statistics of SSA, SSb1, SSb2, and SSD are shown in Figure 3. The results show that the levels of these peaks were significantly changed after processing BC with vinegar (*p* < 0.01), and the contents of SSA and SSD decreased by about 45% and 57%, respectively, while the contents of SSb1 and SSb2 increased by about 17.37 and 26.07 times. The SS composition of BC could be significantly affected by vinegar processing, especially the levels of SSA, SSb1, SSb2, and SSD, but the relationship between this change and the difference in the anti-inflammatory effect had not been further studied.

### 2.2. SSs Effectively Inhibited Pulmonary Edema in LPS-Induced ALI Mice

**Figure 4:**
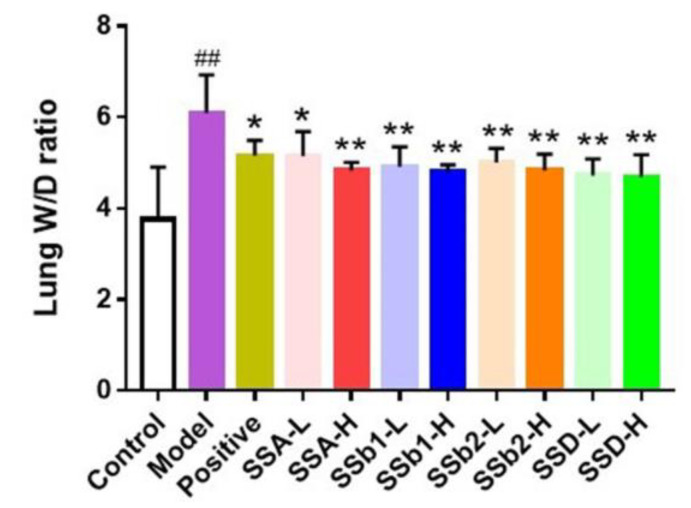
Effects of SSA, SSb1, SSb2, and SSD on lung W/D ratio in LPS-induced ALI mice (*n* = 8, means ± SDs). Analyzed using a one-way ANOVA. ^##^ *p* < 0.01 vs. control group; * *p* < 0.05, ** *p* < 0.01 vs. LPS-induced model group.

As shown in Figure 4, the lung W/D ratio of the LPS-induced ALI group was observably higher than that of the control group (*p* < 0.01), indicating that the ALI model was built successfully. Compared with the LPS-induced ALI group, the lung W/D ratios in the positive, SSA-L, SSA-H, SSb1-L, SSb1-H, SSb2-L, SSb2-H, SSD-L, and SSD-H groups were significantly decreased (*p* < 0.05 or *p* < 0.01). These data indicate that dexamethasone, SSA, SSb1, SSb2, and SSD expressed superior effects for inhibiting pulmonary edema.

### 2.3. SSs Reduced Proinflammatory Cytokine Levels in Serum and Lung Tissue

**Figure 5:**
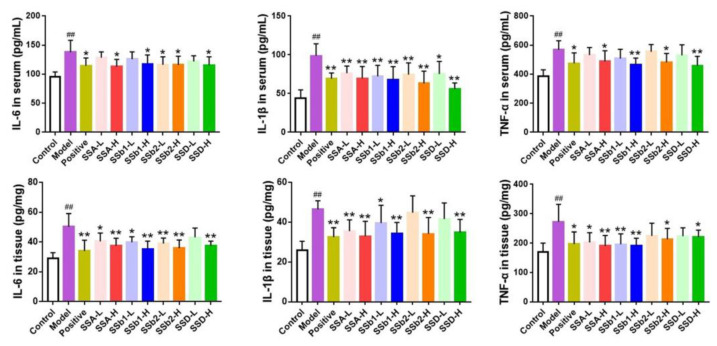
Effects of SSA, SSb1, SSb2, and SSD on the levels of IL-6, IL-1β, and TNF-α in serum and lung tissues from LPS-induced ALI mice (*n* = 8, means ± SDs). Analyzed using a one-way ANOVA. ^##^ *p* < 0.01 vs. control group; * *p* < 0.05, ** *p* < 0.01 vs. LPS-induced model group.

In order to detect the underlying anti-inflammatory activities of SSA, SSb1, SSb2, and SSD in LPS-induced ALI mice, we measured the levels of IL-6, IL-1β, and TNF-α in serum and lung tissues. It was found that severe inflammatory responses clearly occurred in LPS-challenged mice (*p* < 0.01) (Figure 5). The results showed that the increases in IL-6, IL-1β, and TNF-α in serum in the positive, SSA-H, SSb1-H, SSb2-H, and SSD-H groups were markedly restrained (*p* < 0.05 or *p* < 0.01). In addition, compared with the LPS-induced ALI model group, the expressions of IL-6, IL-1β, and TNF-α in lung tissues in the positive, SSA-L, SSA-H, SSb1-L, SSb1-H, SSb2-H, and SSD-H groups were decreased (*p* < 0.05 or *p* < 0.01). In conclusion, SSA, SSb1, SSb2, and SSD had good anti-inflammatory effects and inhibited the expression of the inflammatory factors IL-6, IL-1β, and TNF-α in LPS-challenged ALI mice in a dose-dependent manner.

### 2.4. SSs Relieved the Histological Damage Induced by LPS in Mouse Lungs

**Figure 6:**
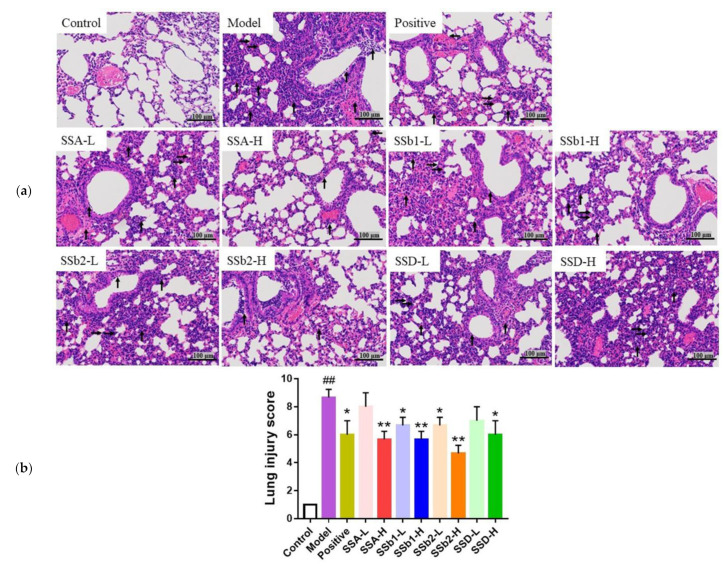
Representative images (**a**) and injury scores (**b**) of H&E-stained lung sections from LPS-induced ALI mice (*n* = 3, means ± SDs). Magnification: ×200. Pathological indices involved in sections: inflammatory (↑), cellular hyperplasia (→), and hemorrhage (←). Analyzed using a one-way ANOVA. ^##^ *p* < 0.01 vs. control group; * *p* < 0.05, ** *p* < 0.01 vs. LPS-induced model group.

The effects of SSA, SSb1, SSb2, and SSD on lung histopathology in LPS-induced ALI mice are showed in Figure 6a,b. The lungs from the control group did not show any pathological injuries, but large amounts of inflammatory cell infiltration, cellular hyperplasia, and hemorrhaging were observed in lungs from the LPS-induced ALI group. When observing and scoring the lung injuries under the microscope, these damages were significantly alleviated in the positive, SSA-H, SSb1-L, SSb1-H, SSb2-L, SSb2-H, and SSD-H groups compared to the LPS-induced group (*p* < 0.05 or *p* < 0.01). In addition, SSb2-H possessed a better protective effect in ALI mice than SSA, SSb1, or SSD.

### 2.5. SSs Restrained the Gene Levels of Inflammatory Cytokines in the Lungs of ALI Mice

**Figure 7:**
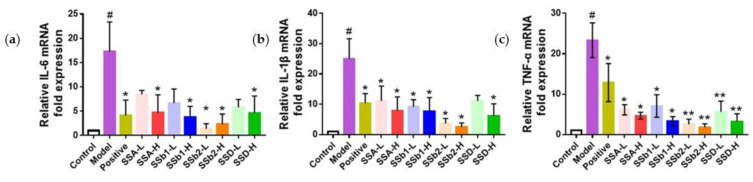
Effects of SSA, SSb1, SSb2, and SSD on mRNA expression levels of IL-6 (**a**), IL-1β (**b**), and TNF-α (**c**) in lung tissues from LPS-induced ALI mice (*n* = 3, means ± SDs). Analyzed using a one-way ANOVA. ^#^ *p* < 0.05 vs. control group; * *p* < 0.05, ** *p* < 0.01 vs. LPS-induced model group.

It was concluded that SSA, SSb1, SSb2, and SSD lowered the levels of IL-6, IL-1β, and TNF-α in serum and lung tissues, exhibiting anti-inflammatory actions on LPS-induced ALI mice. In order to ascertain the anti-inflammatory effects, we investigated the impacts of SSA, SSb1, SSb2, and SSD on the mRNA expression levels of IL-6, IL-1β, and TNF-α in the lungs using RT-qPCR technology. Compared with the control group, the mRNA expression levels of IL-6, IL-1β, and TNF-α in the lungs in the model group were dramatically increased (*p* < 0.05) (Figure 7). However, the mRNA expression levels of IL-6 in the lungs in the positive, SSA-H, SSb1-H, SSb2-L, SSb2-H, and SSD-H groups were inhibited, the IL-1β levels in the lungs in the positive, SSA-L, SSA-H, SSb1-L, SSb1-H, SSb2-L, SSb2-H, and SSD-H groups were reduced, and the TNF-α levels in the lungs in the positive, SSA-L, SSA-H, SSb1-L, SSb1-H, SSb2-L, SSb2-H, SSD-L, and SSD-H groups were decreased (*p* < 0.05 or *p* < 0.01). Therefore, these results demonstrated that SSA, SSb1, SSb2, and SSD caused a dose-dependent inhibition of the mRNA expression of the inflammatory cytokines IL-6, IL-1β, and TNF-α in LPS-induced ALI mice.

### 2.6. SSs Decreased the Expression of TLR4 and NF-κB in the Lungs of ALI Mice

**Figure 8:**
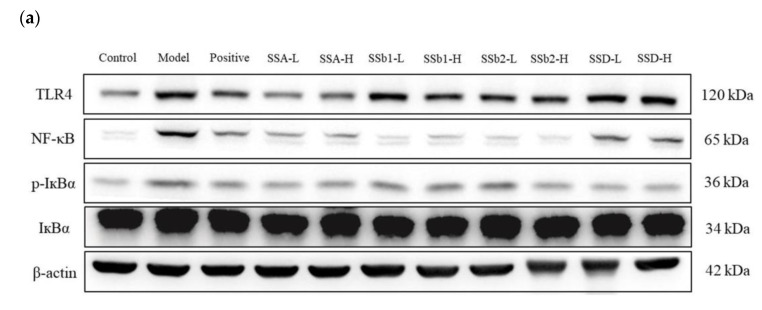

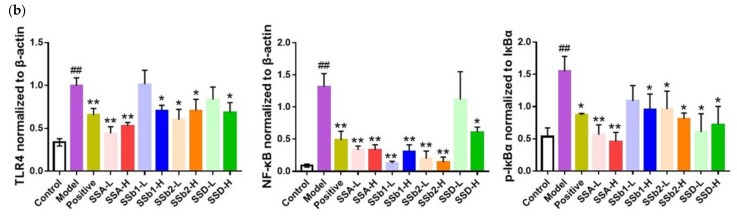
Effects of SSA, SSb1, SSb2, and SSD on protein expression levels of TLR4, NF-κB, p-IκBα, and IκBα in lung tissues from LPS-induced ALI mice (*n* = 3, means ± SDs). (**a**) The expression levels of TLR4, NF-κB, and p-IκBα/IκBα proteins were analyzed using Western blotting. (**b**) The statistical analysis for Western blotting using a one-way ANOVA. ^##^ *p* < 0.01 vs. control group; * *p* < 0.05, ** *p* < 0.01 vs. LPS-induced model group.

In order to further illustrate the anti-inflammatory mechanisms, we surveyed the effects of SSA, SSb1, SSb2, and SSD on the TLR4/NF-κB pathway. As shown in Figure 8a,b, compared with the control group, the protein levels of TLR4 and NF-κB in the LPS-induced ALI group were observably elevated (*p* < 0.01), but the elevations of TLR4 proteins in the positive, SSA-L, SSA-H, SSb1-H, SSb2-L, SSb2-H, and SSD-H groups were lowered, and the increases in NF-κB proteins in the positive, SSA-L, SSA-H, SSb1-L, SSb1-H, SSb2-L, SSb2-H, and SSD-H groups were reduced (*p* < 0.05 or *p* < 0.01). Furthermore, the phosphorylation of the IκBα of NF-κB-related proteins was detected, and the expression levels of p-IκBα/IκBα obviously increased in the ALI group compared to the control group (*p* < 0.01), but the levels of p-IκBα/IκBα in the positive, SSA-L, SSA-H, SSb1-H, SSb2-L, SSb2-H, SSD-L, and SSD-H groups were observably decreased (*p* < 0.05). SSA, SSb1, SSb2, and SSD seemed to effectively inhibit the TLR4/NF-κB pathway.

### 2.7. Statistical Analysis of SS Anti-ALI Indices

In this work, SPSS statistics software was used to standardize the data of fourteen indicators of five types and conduct a principal component analysis. Then, the eigenvalue, variance contributions, and cumulative variance contributions of the principal components of all groups of anti-ALI indicators were obtained. The results are shown in Table 3. The eigenvalues were λ1 = 10.529 and λ2 = 1.135; the respective variance contributions of the two eigenvalues were 75.205% and 8.104%, respectively; and the cumulative variance contribution was 83.309%. In the dimension reduction process of the SPSS principal component analysis, the principal components with eigenvalues ≥1 or cumulative variance contributions >85% were considered to be representative, so they were regarded as the main components. According to the principal component coefficients, the linear combinations of *Y*1 and *Y*2 were obtained. Then, the principal component scores and the comprehensive scores were calculated according to the principal component equation (Formulas (1) and (2)). As showed in Table 4, it could be seen that the comprehensive score results of the pharmacological evaluation system for the anti-ALI efficacy of the different treatment groups were SSb2-H > SSA-H > SSb1-H > SSD-H > positive > SSb2-L > SSA-L > SSb1-L > SSD-L.

*Y*_1_ = 0.286x_1_ + 0.288x_2_ + 0.294x_3_ + 0.264x_4_ + 0.295x_5_ + 0.264x_6_ + 0.271x_7_ + 0.272x_8_ + 0.273x_9_ + 0.281x_10_ + 0.255x_11_ + 0.225x_12_ + 0.233x_13_ + 0.299x_14_, (1)

*Y*_2_ = −0.002x_1_ − 0.138x_2_ − 0.099x_3_ − 0.440x_4_ − 0.099x_5_ − 0.380x_6_ − 0.036x_7_ − 0.284x_8_ + 0.324x_9_ + 0.338x_10_ + 0.457x_11_ − 0.008x_12_ + 0.303x_13_ + 0.145x_14_ (2)

## 3. Discussion

In the Chinese Pharmacopoeia, the contents of SSA and SSD are the indicative components for evaluating the quality of BC or *Bupleurum scorzonerifolium* Willd., but those of SSb1 and SSb2 and their isomers had not been evaluated, especially when the contents of SSb1 and SSb2 were significantly increased after processing with vinegar, and whether their activities are related to structural characteristics had not been studied. Therefore, in this study, we preliminarily investigated the differences in SSs in BC and VBC as well as the protective effects of SSA and its isomers in LPS-induced ALI mice.

Vinegar processing is one of the most important methods in the processing of Chinese herbal medicines, which can greatly reduce the toxicity of toxic herbs, relieve drug properties, increase the cleanliness of herbal medicines, correct odor and taste, enhance the effect on the liver meridian, improve the therapeutic effect, and better prepare for clinical application. Some studies showed that the total SS content was increased after processing BC with vinegar. This may have a certain potential relationship with the enhancement of the role of BC [32]. Multiple studies found that the contents of SSA and SSD declined, while the contents of SSb1and SSb2 increased after being processed with vinegar [13,29]. In this study, we accurately researched the changes in SSs before and after processing with vinegar using UHPLC-Oribtrap/HRMS. The results of the study showed that the contents of SSA and SSD were significantly decreased, but the contents of SSb1 and SSb2 were markedly increased after combining a multivariate statistical analysis with a peak area analysis.

The total SSs alleviated depression-like behaviors and decreased neuroendocrine hormone and inflammatory factor levels by regulating the PI3K/AKT/NF-κB signaling pathway [33]. SSD protected mice from APAP-induced hepatotoxicity, mainly by restraining NF-κB- and STAT3-mediated inflammatory signaling [34]. In other words, the liver protection and antidepressant effects of SSs are closely related to its anti-inflammatory effect. However, it is still unknown whether the anti-inflammatory effects of SSb1 and SSb2 are better than those of SSA and SSD.

In this work, LPS-induced ALI mice were used to investigate the anti-inflammatory effects of SSA, SSb1, SSb2, and SSD and their related mechanisms. Previous research found that, compared with the LPS exposure group, SSA supplementation obviously decreased the lung W/D ratio in a dose-dependent manner [22]. In line with that report, the lung W/D ratio results showed that pulmonary edema in LPS-induced ALI mice was severe, but after SSA, SSb1, SSb2, and SSD treatment, this condition was markedly relieved. The histological analysis showed that polysaccharides from BC significantly ameliorated the pathological damage to lung tissues, with reduced lung edema, restrained alveolar wall thickness, limited inflammatory reactions, and decreased pulmonary hemorrhaging [35]. In our current experiment, the histological results showed that lung injuries such as inflammatory cell infiltration, cellular hyperplasia, and hemorrhaging were visually seen, but these damages were markedly alleviated after SSA, SSb1, SSb2, and SSD treatment. Inflammatory responses in the lungs were triggered by LPS stimulation and were characterized by the outstanding elevation of IL-6, TNF-α, and IL-1β in the serum, BALF, and lung tissue of ALI mice, and these inflammatory factors played an irreplaceable role in mediating immune responses, which could exacerbate tissue and organ damage, so reducing inflammatory responses was deemed to be an effective strategy for the remedy of ALI [36,37,38]. As previously mentioned, we discovered that the degrees of the inflammatory factors TNF-α, IL-6, and IL-1β were clearly increased in the serum and lung tissues of ALI mice, suggesting severe inflammatory lung responses, but under the adjustment of SSA, SSb1, SSb2, and SSD, these rising trends were restrained, indicating that SSA, SSb1, SSb2, and SSD can alleviate lung damage through anti-inflammatory actions. In addition, at the gene expression levels of TNF-α, IL-6, and IL-1β, these results were further verified in lung tissues from ALI mice using an RT-qPCR method.

The expression of NF-κB was closely related to inflammation reactions. The levels of inflammatory factors such as TNF-α, IL-6, and IL-1β were markedly increased after NF-κB was activated by stimuli from the outside [39,40]. Several studies revealed that NF-κB is a vital transcriptional adjuster of inflammatory and immune responses and that the NF-κB signaling pathway plays a significant part in the development of ALI [41,42]. As an important member of the TLR family, Toll-like receptor 4 (TLR4) played a vital role in the inflammatory response, which activated the TLR4/NF-κB signaling pathway and promoted the phosphorylation of IκB after LPS stimulation [43,44]. Our results showed that the expression levels of the NF-κB signaling pathway related proteins TLR4, NF-κB, and p-IκBα/IκBα in ALI model mice were significantly increased, but the levels of those proteins were observably reduced by treatment with SSA, SSb1, SSb2, and SSD. These findings indicated that the protective effects of SSA, SSb1, SSb2, and SSD in LPS-induced ALI mice were mediated by the TLR4/NF-κB signaling pathway.

It has been reported that the structural characteristics of small molecular compounds are closely related to their biological activities. Chenodeoxycholic acid and ursodeoxycholic acid are a pair of epimers. Due to different hydroxy configurations at position 7, ursodeoxycholic acid has stronger hydrophilicity and a unique efficacy in treating cholesterol gallstones. Ursodeoxycholic acid is the only drug approved by the US FDA for the treatment of primary biliary cirrhosis [45]. The structural difference between two ginsenoside stereoisomers could lead to significantly differential physiological activities. Ginsenosides-20(S)-Rg 3 was significantly more potent than ginsenosides-20(R)-Rg 3 on the effects of the agonist PPARγ [46]. SSA and SSD exhibited excellent anti-inflammatory activity via restriction effects on NF-κB activation, but the ether-bond-breaking products SSb1 and SSb2 of SSA and SSD were rarely studied [47]. In this work, the lung W/D ratio, inflammatory factors (IL-6, IL-1β, and TNF-α) in serum and tissue, lung injury scores, and expression levels of genes and proteins associated with the inflammation of LPS-induced ALI mice were first determined. SSA, SSb1, SSb2, and SSD showed excellent lung protection effects. Then, we comprehensively evaluated the lung protection effects using a principal component analysis and found that the effect of SSb2 was the best at the same dose. Meanwhile, we also found that the contents of SSb1 and SSb2 of VBC were dozens of times higher than those of BC. To summarize, we initially believed that SSb1 and SSb2 should also be used as indicators to evaluate the quality of BC, especially the quality of VBC.

## 4. Materials and Methods

### 4.1. Chemicals and Reagents

SSA (A0257), SSb1 (A0260), SSb2 (A0261), and SSD (A0259) were purchased from Chengdu Must Bio-technology Co., Ltd., Chengdu, China. LPS was provided by Sigma Aldrich (Shanghai, China) Trading Co., Ltd., Shanghai, China. TNF-α (RX202412M), IL-1β (RX203049M), and IL-6 (RX203063M) cytokine-specific ELISA kits were obtained from Quanzhou Ruixin Bio-technology Co., Ltd., Quanzhou, China. A Steady Pure Universal RNA Extraction Kit, An *Evo M-MLV* kit, and a SYBR^®^ Green Pro Taq HS premixed kit were provided by AG Accurate Biology Co., Ltd., Changsha, China. Primary antibodies against TLR4 (AF8187), NF-κB (AF1234), p-IκBα (AF1870), IκBα (AF1282), and β-Actin (AF5003) were supplied by Shanghai Beyotime Biotechnology Co., Ltd., Shanghai, Shanghai City of China. All reagents and other chromatographic and analytical-grade chemicals were purchased from local distributors in China.

### 4.2. Preparation and Chemical Analysis of Bupleurum chinense DC.

BC was collected from the Guangzhou Qingping medicinal materials market, Guangdong Province of China, and was distinguished as “*Bupleurum chinense* DC.” by Associate Professor Yuanning Zeng from Guangdong Pharmaceutical University. According to the method of the Chinese Pharmacopoeia, 2020 edition, VBC was prepared, and BC and VBC were powdered and extracted with 10 times the volume in 70% methanol for 30 min under ultrasound [48]. The extracting solutions were concentrated under reduced pressure, and the samples were redissolved with 200 μL volumes in 70% methanol after the solvent was completely volatilized, centrifuged at 13,000 rpm for 10 min, and detected by UHPLC-Oribtrap/HRMS. All samples were tested six times in parallel, and 20 μL of all samples were taken to be used as quality control samples (QC) after mixing. The detection was performed on an Dionex Ultimate 3000 UHPLC system (Thermo Fisher, Waltham, MA, USA) equipped with a Thermo Orbitrap high-resolution mass spectrometer (Thermo Fisher, Waltham, MA, USA). A Waters ACQUITY UPLC HSS T3 column (Waters, Milford, MA, USA) was used for detection at 40 °C, where 0.1% formic acid water and acetonitrile constituted the mobile phase, 0.3 mL/min was set as the flow rate, and the gradient mode was as follows: 0–5 min, 20–50% acetonitrile; 5–13 min, 50–70% acetonitrile; 13–15 min, 70–100% acetonitrile; 15–18 min, 100% acetonitrile; 18–18.10 min, 100–20% acetonitrile; and 18.10–20 min, 20% acetonitrile. In this work, negative ion modes equipped with a thermal spray ionization (HESI) source were used for the qualitative and relative quantitative analyses of SSs. The specific mass spectrometer parameters in the experiment were as follows: spray voltage, 2.80 Kv; ion transfer tube temperature, 300 °C; evaporation temperature, 320 °C; sheath gas, 20 L/min; auxiliary gas, 6 L/min; scanning mode, full MS/ddMS2; scanning range of mass spectrometry, 100~1200 *m*/*z*; resolution, 120,000; and fragmentation energy, 15, 25, and 35. Orbitrap Fusion Tune software and Xcalibur 4.1 software were used for mass spectrum control and data collection.

### 4.3. Establishment of ALI and Treatment

In this study, 8-week-old male BALB/c mice (22–25 g) were purchased from Guangdong Medical Laboratory Animal Center (GDMLAC) (SCXK2022-0002); fed at a humidity of 50 ± 10%, 12/12 h light/dark, and a temperature of 24 ± 1 °C; and given free access to water and food. The experiments were performed according to the rules for the use of the laboratory animals issued by the GDMLAC and were approved by the Animal Ethics Committee of GDMLAC (approval number: C202204–04). The 88 mice were randomly divided into 11 groups (*n* = 8), including a control group, model group, dexamethasone group (5 mg/kg), low-dose SSA group (2.5 mg/kg), high-dose SSA group (10 mg/kg), low-dose SSb1 group (2.5 mg/kg), high-dose SSb1 group (10 mg/kg), low-dose SSb2 group (2.5 mg/kg), high-dose SSb2 group (10 mg/kg), low-dose SSD group (2.5 mg/kg), and high-dose SSD group (10 mg/kg). One hour after the last administration, the ALI model was established by instilling intratracheally with LPS (5 mg/kg), and the control group was instilled with PBS [49,50]. The dexamethasone, SSA, SSb1, SSb2, and SSD groups were intraperitoneally given the drugs once per day for 7 consecutive days before the establishment of the model. The control groups and model groups were offered normal saline. After twelve hours of LPS challenge, the blood and lung tissues were collected and stored at −80 °C in a fridge for subsequent tests.

### 4.4. The Lung W/D Ratio Analysis

The degrees of pulmonary edema were assessed via the lung W/D ratio [51]. The wet weight of each lung from all groups was measured and recorded after intrapulmonary blood was completely removed. Then, these lung tissues were dried in an oven at 60 °C for 48 h until the weight no longer changed. Ultimately, the lung wet and dry (W/D) ratio was calculated and analyzed.

### 4.5. Proinflammatory Cytokine Levels Analysis

The TNF-α, IL-6, and IL-1β levels in serum and lung tissues were measured using ELISA kits according to the manufacturer’s instructions. The lung tissues from all groups were homogenized with 0.9% saline (1:9, g/L) to prepare a lung tissue homogenization buffer and were centrifuged at −4 °C and 12,000 rpm for 10 min. Then, the supernatant was collected, the levels of proinflammatory cytokines were measured, and the protein concentrations in the lung tissue supernatant were quantified using a BCA Protein Assay Kit.

### 4.6. Histopathological Analysis 

After the mice from all groups were sacrificed, partial lung tissues were collected and fixed in a 4% paraformaldehyde (pH 7.4) solution. Then, the lung tissues were dehydrated, embedded, sectioned, and stained with hematoxylin and eosin (H&E). The pathological changes in the lung tissues were evaluated using a light microscope (Nikon, Tokyo, Japan, Eclipse Ci-L). According to a previous study [52], lung injury scores were determined to display inflammatory, cellular hyperplasia, and hemorrhage by grading injuries as severe (4), moderate (3), mild (2), very slight (1), or nil (0).

### 4.7. Real-Time Quantitative PCR Analysis

The total RNA from murine lung tissues was isolated, transcribed, quantified, and measured using a real-time fluorescent quantitative PCR instrument. After normalizing the 18S gene as a control, the mRNA expression levels of the lung tissues were detected. All real-time quantitative PCR (RT-qPCR) procedures were accomplished according to the manufacturer’s instructions. Detailed primer sequences are listed in Table 5.

### 4.8. Western Blotting Analysis

The lung tissues were collected, weighed, and added to a radio immunoprecipitation assay (RIPA) lysis buffer in proportion. Then, the samples were homogenized using a low-temperature tissue grinder (SCIENTZ-48L, Scientz, Ningbo, China), placed on ice for 20 min, and centrifuged at 13,000 rpm for 30 min to collect the supernatant. After the protein concentrations of the supernatants were determined using a BCA assay kit, 40 μg of protein per sample was proportionally blended with a loading buffer and denatured in a boiling water bath for 10 min. The proteins of the lung tissues were separated, transferred, and correspondingly incubated overnight with primary antibodies against TLR4, NF-κB, p-IκBα, IκBα, and β-Actin at 4 °C, followed by incubation with an HRP-labeled goat anti-rabbit secondary antibody at 37 °C for 60 min. The proteins were then detected, photographed, and quantified using a Monad chemiluminescence imaging system (GD50202, Monad Biotech, Suzhou, China).

### 4.9. Principal Component Analysis

SPSS statistical software was used to conduct a principal component analysis on the results of all indicators, and the principal component eigenvalue, cumulative variance contribution, and principal component score were calculated.

### 4.10. Statistical Analysis

All data were analyzed using GraphPad Prism 8.0. Measurement data were expressed as means ± SDs. Statistical significance was analyzed using a one-way ANOVA, and *p* < 0.05 was considered statistically significant.

## 5. Conclusions

In conclusion, we first used a UHPLC-Orbitrap/HRMS method to determine the differences in chemical composition between BC and VBC and revealed that the contents of SSb1 and SSb2 were markedly increased after processing with vinegar. We also confirmed the anti-inflammatory effects and possible mechanisms of SSA, SSb1, SSb2, and SSD in LPS-induced ALI mice. Moreover, the lung protection effects of SSb2 were better than the others at the same dose. These results could effectively explain the potential principle of the efficacy enhancement method of vinegar processing. Of course, we also suggest that SSb2 and SSb1 should also were used as indicators of quality control for VBC.

## Figures and Tables

**Table 1:** Chromatographic retention time and mass spectrum information of the identified compounds.

Number	Identification	RT (min)	Molecular Formula	Theoretical [M-H]^−^	Experimental [M-H]^−^	Error (ppm)	MS/MS
1	Chlorogenic acid	2.42	C_16_H_18_O_9_	353.08671	353.08444	6.425	353.08444, 191.05335
2	Rutin	3.27	C_27_H_30_O_16_	609.14501	609.13983	8.506	609.13983, 300.02368
3	Narcissoside	4.07	C_28_H_32_O_16_	623.16066	623.15674	6.292	623.15674, 315.04791, 314.04306
4	Bupleuroside V	4.74	C_42_H_66_O_15_	809.43180	809.42487	8.559	855.42505, 809.42487, 779.41461, 617.36328, 471.30539
5	epinortrachelogenin	5.99	C_20_H_22_O_7_	373.12818	373.12521	7.958	373.12521, 179.06854, 164.04540
6	Quercetin	6.01	C_15_H_10_O_7_	301.03428	301.03262	5.511	301.03262, 151.00133
7	Chikusaikoside II	6.39	C_48_H_78_O_18_	941.51044	941.50360	7.267	987.50659, 941.50360, 795.44507, 779.45148, 617.40125
8	Kaempferol	6.86	C_15_H_10_O_6_	285.03936	285.03812	4.366	285.03812, 151.07874
9	HO-SSc	7.08	C_48_H_80_O_18_	943.52609	943.52014	6.308	989.51270, 943.52014, 797.44751
10	SSf	7.10	C_48_H_79_O_17_	927.53118	927.52325	8.547	973.52899, 927.52325, 781.46655, 765.47333, 619.41663
11	Isorhamnetin	7.11	C_16_H_12_O_7_	315.05775	315.04709	9.012	315.047089, 300.02390, 151.00102
12	SSc	7.26	C_48_H_78_O_17_	925.51553	925.50879	7.279	971.51337, 925.50879, 779.45245, 763.45630, 617.40051
13	SSb3	7.68	C_43_H_72_O_14_	811.48383	811.47858	6.474	857.48779, 811.47858, 649.42609, 471.34198
14	SSb4	7.80	C_43_H_72_O_14_	811.48383	811.47821	6.929	857.48645, 811.47821, 649.42554
15	Acetyl-SSc	7.84	C_50_H_80_O_18_	967.52609	967.51984	6.462	1013.52161, 967.51984, 925.50836, 907.49945, 779.44580, 761.43976
16	Acetyl-SSb3	8.21	C_45_H_74_O_15_	853.49440	853.48871	6.664	899.49365, 853.48871, 811.47876, 793.46765, 649.42651
17	SSA	8.39	C_42_H_68_O_13_	779.45762	779.45154	7.798	825.45807, 779.45154, 617.40009, 471.34628
18	SSb2	8.75	C_42_H_68_O_13_	779.45762	779.45184	7.413	825.45422, 779.45184, 617.39990, 471.34164
19	2″-*O*-acetyl-SSA	8.79	C_44_H_70_O_14_	821.46818	821.46210	7.405	821.46210, 779.45355, 761.44025, 617.39899
20	SSb1	8.87	C_42_H_68_O_13_	779.45762	779.45215	7.016	825.45758, 779.45215, 617.40082, 471.34387
21	2″-*O*-acetyl-SSb2	8.91	C_44_H_70_O_14_	821.46818	821.46442	4.581	821.46442, 779.45215, 761.45129, 617.40057
22	Acetyl-SSb4	8.96	C_45_H_74_O_15_	853.49440	853.48908	6.242	899.49561, 853.48908, 811.47858, 793.46942, 649.42645
23	3″-*O*-acetyl-SSA	9.03	C_44_H_70_O_14_	821.46818	821.46625	2.353	821.46625, 779.45239, 761.43872
24	Prosaikogenin H	9.28	C_36_H_58_O_8_	617.40480	617.40076	6.536	663.40533, 617.40076
25	Prosaikogenin F	9.52	C_36_H_58_O_8_	617.40480	617.40125	5.742	663.40643, 617.40125
26	SSe	9.54	C_42_H_68_O_12_	763.46270	763.45752	6.790	809.46307, 763.45752, 601.40729
27	SSm	9.67	C_42_H_68_O_12_	763.46270	763.45691	7.589	809.46191, 763.45691, 601.40564
28	3″-*O*-acetyl-SSb2	9.68	C_44_H_70_O_14_	821.46818	821.46161	8.002	821.46161, 779.45032, 617.39728
29	2″-*O*-acetyl-SSb1	9.78	C_44_H_70_O_14_	821.46818	821.46204	7.478	821.46204, 779.46057, 617.40015
30	3″-*O*-acetyl-SSb1	9.88	C_44_H_70_O_14_	821.46818	821.46167	7.929	821.46167, 779.44995, 617.40662
31	4″-*O*-acetyl-SSb1	10.33	C_44_H_70_O_14_	821.46818	821.46649	2.061	821.46649, 617.39783
32	Diacetyl-SSb2	10.40	C_46_H_72_O_15_	863.47875	863.47522	4.086	909.48029, 863.47522, 821.45264, 761.44074
33	2″-*O*-acetyl-SSe	10.57	C_44_H_70_O_13_	805.47327	805.46771	6.901	851.47345, 805.46771, 763.45740, 745.44678, 601.40863
34	6″-*O*-acetyl-SSb1	10.60	C_44_H_70_O_14_	821.46818	821.46393	5.177	821.46393, 779.45471
35	SSD	10.66	C_42_H_68_O_13_	779.45762	779.45258	6.464	825.45654, 779.45258, 617.40076
36	2″-*O*-acetyl-SSm	10.93	C_44_H_70_O_13_	805.47327	805.46710	7.658	851.47400, 805.46710, 763.45471, 745.44263
37	SSY	11.08	C_42_H_66_O_13_	777.44197	777.43640	7.162	823.44617, 777.43640, 615.38428
38	4″-*O*-acetyl-SSA	11.20	C_44_H_70_O_14_	821.46818	821.46240	7.040	821.46240, 779.45435, 761.44324, 617.39819
39	4″-*O*-acetyl-SSb2	11.32	C_44_H_70_O_14_	821.46818	821.46619	2.426	821.46619, 779.44183, 761.45038, 617.40247
40	6″-*O*-acetyl-SSA	11.44	C_44_H_70_O_14_	821.46818	821.46100	8.744	821.46100, 779.44879
41	6″-*O*-acetyl-SSb2	11.58	C_44_H_70_O_14_	821.46818	821.46167	7.929	821.46167, 779.44751
42	3″-*O*-acetyl-SSe	11.66	C_44_H_70_O_13_	805.47327	805.46698	7.807	851.47260, 805.46698, 763.45648, 745.44745, 601.40564
43	2″-*O*-acetyl-SSD	11.70	C_44_H_70_O_14_	821.46818	821.46289	6.443	821.46289, 779.45270, 761.44043, 617.39905
44	3″-*O*-acetyl-SSD	11.82	C_44_H_70_O_14_	821.46818	821.46228	7.186	821.46228, 779.45050, 761.44135
45	3″-*O*-acetyl-SSm	12.04	C_44_H_70_O_13_	805.47327	805.46649	8.416	851.47028, 805.46649, 763.45490, 745.44727
46	Prosaikogenin G	12.47	C_36_H_58_O_8_	617.40480	617.40000	7.831	663.40503, 617.40000
47	4″-*O*-acetyl-SSD	12.59	C_44_H_70_O_14_	821.46818	821.45367	7.668	821.45367, 779.45801, 617.39990
48	6″-*O*-acetyl-SSD	12.70	C_44_H_70_O_14_	821.46818	821.46143	8.221	821.46143, 617.40204
49	Diacetyl-SSA	12.93	C_46_H_72_O_15_	863.47875	863.47205	7.757	909.47742, 863.47205, 821.46124, 779.45111, 761.43970, 617.39887
50	6″-*O*-acetyl-SSe	13.96	C_44_H_70_O_13_	805.47327	805.46552	9.620	851.47376, 805.46552, 763.45001, 745.44611
51	Diacetyl-SSD	13.68	C_46_H_72_O_15_	863.47875	863.47089	9.100	909.47644, 863.47089, 821.46143, 761.43646, 617.39813
52	6″-*O*-acetyl-SSm	14.90	C_44_H_70_O_13_	805.47327	805.46491	6.010	851.47363, 805.46491, 763.45136, 745.44421

SS: saikosaponin.

**Table 2:** The characterized compounds of BC changed after processing.

Number	Identification	VIP	*p*-Value	Change Trend after Processing
1	Diacetyl-SSb2	1.28867	<0.001	↑
2	SSb1	1.2881	<0.001	↑
3	Acetyl-SSc	1.27938	<0.001	↑
4	6″-*O*-acetyl-SSb1	1.27844	<0.001	↑
5	2″-*O*-acetyl-SSD	1.27832	<0.001	↓
6	6″-*O*-acetyl-SSb2	1.27645	<0.001	↓
7	4″-*O*-acetyl-SSA	1.27386	<0.001	↓
8	Prosaikogenin H	1.27353	<0.001	↑
9	SSm	1.27284	<0.001	↑
10	2″-*O*-acetyl-SSA	1.26759	<0.001	↓
11	3″-*O*-acetyl-SSA	1.25863	<0.001	↓
12	3″-*O*-acetyl-SSe	1.25478	<0.001	↑
13	4″-*O*-acetyl-SSb2	1.24614	<0.001	↓
14	SSb2	1.24421	<0.001	↑
15	SSA	1.24241	<0.001	↓
16	SSD	1.24104	<0.001	↓
17	4″-*O*-acetyl-SSb1	1.24072	<0.001	↑
18	6″-*O*-acetyl-SSA	1.23792	<0.001	↓
19	3″-*O*-acetyl-SSm	1.23202	<0.001	↑
20	6″-*O*-acetyl-SSe	1.23135	<0.001	↓
21	2″-*O*-acetyl-SSb1	1.22735	<0.001	↑
22	Prosaikogenin G	1.22414	<0.001	↓
23	3″-*O*-acetyl-SSb1	1.18866	<0.001	↑
24	Rutin	1.16195	<0.001	↓
25	Diacetyl-SSA	1.15538	<0.001	↓
26	2″-*O*-acetyl-SSe	1.05265	0.01 < *p* < 0.001	↓

SS: saikosaponin; “↑” and “↓” means rising and decline after processing with vinegar.

**Table 3:** The eigenvalues and variance contributions of the principal components.

Ingredient	Initial Eigenvalue			Extract Square Sum Load		
	Total	Variance Contribution (%)	Cumulative Variance Contribution (%)	Total	Variance Contribution (%)	Cumulative Variance Contribution (%)
1	10.529	75.205	75.205	10.529	75.205	75.205
2	1.135	8.104	83.309	1.135	8.104	83.309

**Table 4:** The principal component scores and comprehensive scores.

Group	Dosage (mg/kg)	Score of Principal Components *Y*_1_	Score of Principal Components *Y*_2_	Comprehensive Score	Number
Control	--	−6.00	1.52	−4.39	1
Model	--	7.69	1.45	5.90	11
Positive	5.00	−0.36	1.06	−0.19	6
SSA-L	2.50	0.76	−0.63	0.52	8
SSA-H	10.00	−1.33	−0.03	−1.00	3
SSb1-L	2.50	0.97	−0.50	0.69	9
SSb1-H	10.00	−1.03	0.12	−0.76	4
SSb2-L	2.50	0.29	−2.13	0.05	7
SSb2-H	10.00	−1.55	−0.59	−1.22	2
SSD-L	2.50	1.53	−0.47	1.12	10
SSD-H	10.00	−0.97	0.20	−0.72	5

SS: saikosaponin; “--” stands for giving normal saline.

**Table 5:** The sequences of primers for TNF-α, IL-1β, IL-6, and 18S used in this study.

Gene Name	Forward Primers (5′–3′)	Reverse Primers (5′–3′)
TNF-α	ATGTCTCAGCCTCTTCTCATTC	GCTTGTCACTCGAATTTTGAGA
IL-1β	TGGTGTGTGACGTTCCCATT	CAGCACGAGGGTTTTTTGTTG
IL-6	CCGGAGAGGAGACTTCACAG	CAGAATTGCCATTGCACAAC
18S	AGTTCCAGCACATTTTGCGAG	TCATCCTCCGTGAGTTCTCCA

TNF-α, tumor necrosis factor α; IL-1β, interleukin 1β; IL-6, interleukin 6.

## Data Availability

The data supporting the findings of the present study are not publicly available. The data are available only upon reasonable request and with permission from the authors.
